# Acute gastric dilatation in a patient with anorexia nervosa binge/purge subtype

**DOI:** 10.4103/0974-2700.70774

**Published:** 2010

**Authors:** Ailis M Tweed-Kent, Peter J Fagenholz, Hasan B Alam

**Affiliations:** Division of Trauma, Emergency Surgery, and Critical Care, Department of Surgery, Massachusetts General Hospital, 55 Fruit St., Boston, MA, USA

**Keywords:** Acute gastric dilatation, anorexia, binge/purge, denial, eating disorder, gastric dysmotility

## Abstract

Acute gastric dilatation is a rare complication of anorexia nervosa binge/purge subtype that results from gastrointestinal abnormalities, including decreased gastric motility and delayed gastric emptying. Early diagnosis and intervention is critical since delay may result in gastric necrosis, perforation, shock, and death. We report a 26-year-old female with anorexia nervosa binge/purge subtype, who presented with abdominal pain and nausea after a binge episode. Abdominal radiography and computed tomography showed a grossly dilated stomach measuring 32 cm × 17.9 cm consistent with acute gastric dilatation. She underwent exploratory laparotomy with gastrotomy and gastric decompression, and recovered uneventfully. Initially, the patient denied the binge episode, as many patients with eating disorders do, but later revealed an extensive history of anorexia nervosa binge/purge subtype. This case stresses the importance of obtaining a thorough history of eating disorders and maintaining a high index of suspicion for acute gastric dilatation in young women who present with abdominal pain and distention.

## INTRODUCTION

Anorexia nervosa binge/purge subtype is a disorder characterized by a refusal to maintain adequate body weight with intermittent binge/purge episodes. Anorexia nervosa and bulimia nervosa afflict about 0.6–1% of the population of USA.[1] Both the conditions can cause chronic gastrointestinal abnormalities, including decreased gastric motility and delayed gastric emptying, which may rarely lead to acute gastric dilatation.[2] This is considered a surgical emergency as gastric necrosis, perforation, shock, and death can occur with delayed treatment.[3–5] Nonetheless, acute gastric dilatation related to eating disorders has received insufficient attention in the literature, given the prevalence of eating disorders, the frequent reluctance of patients to divulge these conditions, and the serious consequences of delay in diagnosis and treatment.[6] We present here a case of acute gastric dilatation requiring urgent laparotomy in a patient with anorexia nervosa binge/purge subtype, and review the difficulty of obtaining an accurate history and the good functional outcomes associated with early intervention.

## CASE REPORT

A 26-year-old female with a history of anxiety and depression presented to our Emergency Department with sudden onset of diffuse abdominal pain, nausea, and an inability to vomit of 2 hours duration. Her pain was sharp, severe, and worst in the epigastrium. She reported drinking four beers and eating a Cobb salad several hours prior to presentation. She denied diarrhea, constipation, fever, and chills. She reported no similar episodes in the past and denied the consumption of non-food items.

Physical examination revealed a thin, uncomfortable appearing young woman. Her weight was 99 pounds and height was 61 inches. Her temperature was 98.6°F, pulse was 100 beats per minute, and blood pressure was 114/77 mm Hg. The abdomen was distended, firm, and diffusely tender to palpation without peritonitis. All other examination findings were non-contributory.

**Figure 1:**
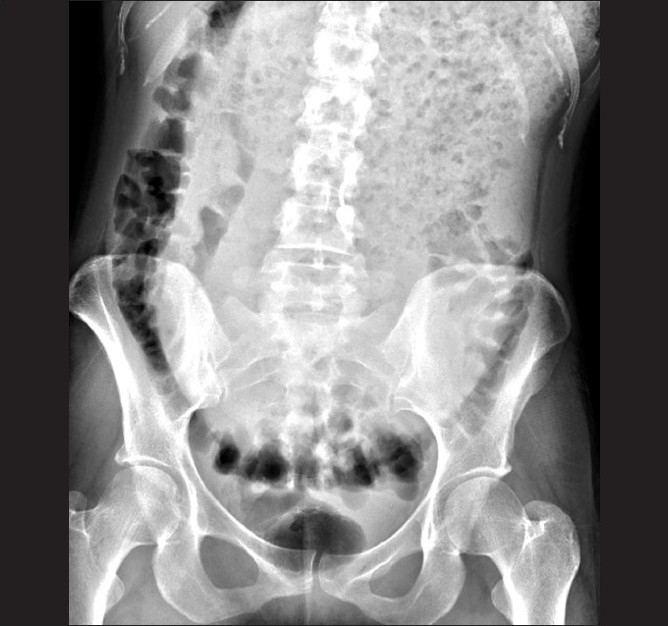
Abdominal radiograph showing a massively enlarged stomach, which occupies the entire abdominal cavity and displaces the transverse colon into the pelvis

**Figure 2:**
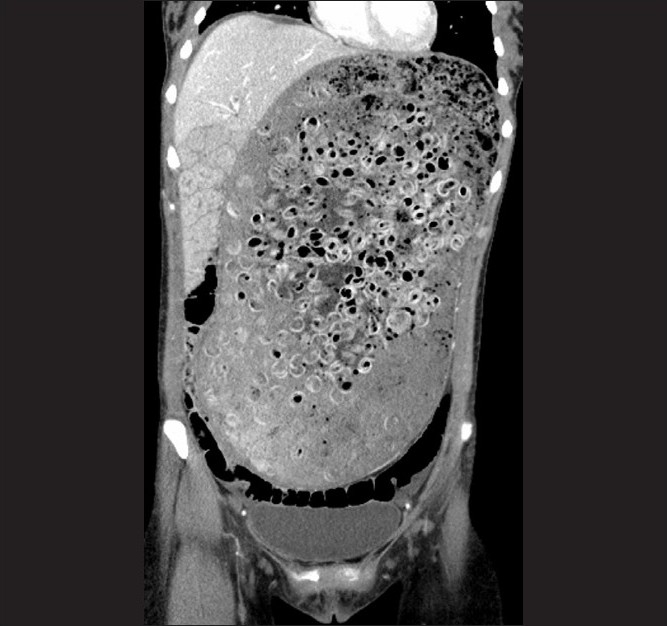
Coronal image of an abdominal CT scan, which reveal a distended stomach containing air and circular radiodense material, which was later revealed to be pasta

Laboratory abnormalities included potassium 3.1 mEq/L, amylase 210 U/L, and lipase 114 U/L. Remaining chemistries and a complete blood count were normal. Abdominal plain film showed a grossly distended stomach filled with extensive food matter [Figure 1]. There was no abdominal free air. Abdominal computed tomography (CT) demonstrated an enlarged stomach, measuring 32 cm × 17.9 cm, displacing the small bowel into the right lower quadrant and the transverse colon into the pelvis [Figure 2].

The patient underwent a laparotomy (through a small epigastric midline incision) which revealed a massively distended stomach. Anterior gastrotomy showed that the stomach was filled with nearly 2 gallons of partially and minimally digested food including whole pasta shells and vegetable matter. Due to the large size and thick consistency of the stomach contents, they were removed via a 40-Fr. chest tube secured in the gastrotomy site, with gentle massage of stomach contents through the tube into a basin. Following adequate decompression, the tube was removed, the stomach mobilized out of the wound, the remaining contents emptied, and the gastrotomy closed. No discrete bezoar or mechanical gastric outlet obstruction was identified. Of note, the gastric wall was poorly perfused due to the massive distention, and the site of gastrotomy showed patchy necrosis even after decompression. Thus, a portion of the anterior wall was resected to viable appearing tissues to allow for safe closure of the gastrotomy. Pathology of the excised gastric wall showed gastric mucosal necrosis.

The following day, the patient admitted to a massive 1-hour binge prior to presentation, and revealed a history of anorexia nervosa and binge eating. At age 14, she had been diagnosed with anorexia nervosa binge/purge subtype. Although she had not engaged in binge eating for 4 years, she attributed this episode to alcohol intoxication and stress.

Postoperatively, a nasogastric tube was left in place for gastric decompression, and a post-pyloric feeding tube in case of gastric ileus after the extreme distension. The nasogastric tube was removed on day 3 and a clear liquid diet started. She advanced to a full liquid diet and was discharged on day 5. She has since resumed a full diet and has reported no problems.

## DISCUSSION

Acute gastric dilatation with necrosis is a rare complication of binge/purge subtype anorexia nervosa.[4,5] There are numerous other causes of acute gastric distention, including resumption of feeding after starvation, diabetes mellitus, bezoars, gastrointestinal tumors, superior mesenteric artery syndrome, ulcers, tumors, gastric volvulus, annular pancreas, gastroduodenal tuberculosis, gastroduodenal Crohn’s disease, etc.[7–10] Patients with anorexia nervosa and bulimia nervosa, approximately 60% of whom will have gastric dysmotility, are at increased risk for acute gastric dilatation due to decreased gastric motility, increased gastric capacity, and decreased gastric emptying.[2,11] Even years after the cessation of anorexia nervosa, patients may still have gastric dysmotility, predisposing any patient with a history of an eating disorder to acute gastric dilatation.[2]

The pathophysiology of gastric dysfunction in anorexia nervosa binge/purge subtype is poorly understood. Possible mechanisms include gastrointestinal smooth muscle atrophy, diminished release of cholecystokinin, abnormalities in the autonomic nervous system, and gastric rhythm abnormalities.[2]

Patients with acute gastric dilatation typically present with sudden onset abdominal pain, distention, and inability to vomit. It is important to elicit any present or remote history of eating disorders, which patients may initially deny.[6] Maintaining a high index of suspicion for acute gastric dilatation is crucial, since delays in treatment have been associated with high mortality.[3]

Feared complications of acute gastric dilatation include necrosis, perforation, shock, and death.[3–5] As the intragastric pressure rises above 30 cm H_2_O, a decrease in venous outflow may result in ischemia and infarction of the gastric wall which can rupture.[3] The mortality rates associated with gastric wall necrosis and rupture have been reported to be 37.5 and 55.6%, respectively.[5] In our case, the pathology showed mucosal ischemia, demonstrating that the process of gastric infarction was already underway.

Initial treatment of acute gastric dilatation includes nasogastric decompression and fluid resuscitation.[4] However, as in this case, when nasogastric decompression is impossible due to the large retained food items, urgent laparotomy and gastrotomy are required to evacuate the stomach contents. In stable patients, a CT scan or other imaging may be useful to assess the feasibility of NGT decompression and to determine the etiology of the gastric outlet obstruction. However, unstable patients with suspected acute gastric dilatation should undergo immediate diagnostic and therapeutic laparotomy without delay for imaging.

## CONCLUSION

In conclusion, this case report stresses the importance of a thorough history of eating disorders in young women who present with distention and abdominal pain; a high index of suspicion should remain even when the patient denies an eating disorder. If acute gastric dilatation is suspected and non-operative treatment is not viable, early surgical intervention is critical in preventing fatal complications.

## References

[1] Hudson JI, Hiripi E, Pope HG, Kessler RC (2007). The prevalence and correlates of eating disorders in the National Comorbidity Survey Replication. Biol Psychiatry.

[2] Hadley SJ, Walsh BT (2003). Gastrointestinal disturbances in anorexia nervosa and bulimia nervosa. Curr Drug Targets CNS Neurol Disord.

[3] Turan M, Sen M, Canbay E, Karadayi K, Yildiz E (2003). Gastric necrosis and perforation caused by acute gastric dilatation: report of a case. Surg Today.

[4] Nakao A, Isozaki H, Iwagaki H, Kanagawa T, Takakura N, Tanaka N (2000). Gastric perforation caused by a bulimic attack in an anorexia nervosa patient: report of a case. Surg Today.

[5] Watanabe S, Terazawa K, Asari M, Matsubara K, Shiono H, Shimizu K (2008). An autopsy case of sudden death due to acute gastric dilatation without rupture. Forensic Sci Int.

[6] Vandereycken W, Van Humbeeck I (2008). Denial and concealment of eating disorders: a retrospective survey. Eur Eat Disord Rev.

[7] Franken EA, Fox M, Smith JA, Smith WL (1978). Acute gastric dilatation in neglected children. AJR Am J Roentgenol.

[8] Nagai T, Yokoo M, Tomizawa T, Mori M (2001). Acute gastric dilatation accompanied by diabetes mellitus. Intern Med.

[9] Sehgal VN, Srivastava G (2006). Trichotillomania ± trichobezoar: revisited. J Eur Acad Dermatol Venereol.

[10] Kusunoki M, Hatada T, Ikeuchi H, Okamoto T, Sakanoue Y, Utsunomiya J (1992). Gastric volvulus complicating myotonic dystrophy. Hepatogastroenterology.

[11] Benini L, Todesco T, Dalle Grave R, Deiorio F, Salandini L, Vantini I (2004). Gastric emptying in patients with restricting and binge/purging subtypes of anorexia nervosa. Am J Gastroenterol.

